# A novel strategy to facilitate uniform epithelial cell maturation using liquid–liquid interfaces

**DOI:** 10.1038/s41598-024-63115-7

**Published:** 2024-05-29

**Authors:** Rie Sonoi, Masamichi Kamihira

**Affiliations:** Department of Chemical Engineering, Faculty of Engineering, Kyushu University, 744 Motooka, Nishi-Ku, Fukuoka, 819-0395 Japan

**Keywords:** Liquid–liquid interfaces, Epithelial cells, Tight junction, Uniform maturation, Nucleus density, Cell behavior, Biophysics, Biotechnology, Cell biology, Engineering, Materials science, Biomaterials

## Abstract

Epithelial tissue forms and maintains a critical barrier function in the body. A novel culture design aimed at promoting uniform maturation of epithelial cells using liquid materials is described. Culturing Madin–Darby canine kidney (MDCK) cells at the liquid–liquid interface yielded reduced migration and stimulated active cell growth. Similar to solid–liquid interfaces, cells cultured on a fibronectin-coated liquid–liquid interface exhibited active migration and growth, ultimately reaching a confluent state. These cells exhibited reduced stress fiber formation and adopted a cobblestone-like shape, which led to their even distribution in the culture vessel. To inhibit stress fiber formation and apoptosis, the exposure of cells on liquid–liquid interfaces to Y27632, a specific inhibitor of the Rho-associated protein kinase (ROCK), facilitated tight junction formation (frequency of ZO-2-positive cells, *F*_Z_ = 0.73). In Y27632-exposed cells on the liquid–liquid interface, the value obtained by subtracting the standard deviation of the ratio of nucleus densities in each region that compartmentalized a culture vessel from 1, denoted as *H*_LN_, was 0.93 ± 0.01, indicated even cell distribution in the culture vessel at *t* = 72 h. The behavior of epithelial cells on liquid–liquid interfaces contributes to the promotion of their uniform maturation.

## Introduction

Liquid–liquid interfaces naturally exist in living organisms, such as in the gastric mucosa and the tear fluid of the eye, providing an important environment for functional maturation. Recently, as a strategy for corneal repair, Hung et al.^1^ have reported the establishment of a method for forming tissue-like structures using the liquid–liquid interface of an aqueous two-phase system, consisting mainly of polyethylene glycol and dextran. Liquid–liquid interfaces in vitro, the interface between two immiscible phases, is an attractive tool for research in materials science and tissue engineering applications^2–7^. Although solid materials such as polystyrene are used from the standpoint of cell culture manipulation, it is possible to grow anchorage-dependent cells at the phase boundary between a fluorocarbon fluid and the culture medium^2–7^. Keese and Giaever^2^ have proposed a method for culturing cells on a nanosheet formed by the addition of small quantities of pentafluorobenzoyl chloride to alumina-treated fluorocarbon fluids. Kong et al.^3–5^ have proposed methods enabling cell adhesion to liquid interfaces for long-term culture. These methods involve the fabrication of nanosheets on perfluorocarbon (PFC), with cell growth reportedly linked to the viscoelastic profile of protein nanosheets^3–5^. Furthermore, it has been reported that mechanical force from liquid scaffolds is an important event to guide the fate of mesenchymal stem cells^2–7^. Thus, controlled cell behavior from the liquid–liquid interface is possible and understanding their behavior such as growth and migration in accordance with various forces from the external environment on the cell may lead to novel culture design for the uniform maturation of epithelial cells.

Cultivating matured epithelial cells at high density is crucial for preserving polarity and function. Subsequently, the cells regulate their behavior and characteristics, responding to their microenvironment through both cell–cell and cell–matrix adhesions^8,9^. The formation and maintenance of tight junctions (TJs), which facilitate cell binding, play an indispensable role in the maturation process; the cultivation method is employed to induce polarized epithelial cells. In confluent cultures, the behavior of epithelial cells is altered in response to the level of cell–cell adhesion^10–13^, suggesting that the maturation is influenced by behaviors such as cell growth and migration. Understanding the behavior and characteristics of the epithelial cells in a confluent state through analyses based on the concepts of position and time is an important strategy for achieving uniform maturation in a culture vessel.

The actin cytoskeleton interacts with adaptor proteins, such as zonula occludens-2 (ZO-2), to form TJs and binds to the TJ proteins via ZO-2 binding to the TJ localized at the cellular membrane^14^. These interactions between TJs and the actin cytoskeleton are essential for the maintenance of TJ integrity and polarity. In accordance with cell behavior, ZO-2 undergoes changes in its subcellular localization depending on the degree of cell maturation, translocating to the cell nucleus during active cell growth and to the plasma membrane during TJ formation^15,16^. The differences in the subcellular localization of ZO-2 elucidate the extent of maturation. Furthermore, the assembly and disassembly of the actin cytoskeleton are regulated by RhoA, Rac1, and Cdc42 of the Rho small GTPase family. Rho and Rac, play a pivotal role in regulating actin cytoskeleton remodeling, survival, migration, and growth in epithelial cells^17–19^. In the maintenance of TJs, the assembly and disassembly of the actin cytoskeleton at TJs appears to be coordinately controlled by the Rho and Rac. In this study, MDCK cells cultured on a non-coated liquid–liquid interface using a specific inhibitor for Rho-dependent protein kinase (ROCK), i.e., endogenous stimulus approach, or a fibronectin-coated liquid–liquid interface, i.e., exogenous stimulus approach, were subjected to time-lapse imaging and immunostaining, followed by the analysis of the association between cell behavior and the extent of TJ formation during maturation in the culture vessel; uniform maturation was attempted.

## Methods

### Preparation of liquid–liquid interfaces

In this study, we focused on interfaces formed with the fluorinated oil Novec-7500 (F051243; Fluorochem, UK), owing to its broad application in various microdroplet microfluidic technologies and its low cytotoxicity. To each well of 24-well plates (3526; Corning, NY, USA), 1 mL Novec-7500 was added (Fig. S1A). A 20 μg/mL fibronectin solution (1918-FN-02M; R&D Systems, Minneapolis, MN, USA) in phosphate-buffered saline (PBS) was slowly introduced into the culture vessel and incubated for 1 h at room temperature (32–37 °C), and then washed four-times with PBS. Subsequently, the surfaces were washed with E-MEM and replaced with E-MEM containing 10% FBS (173012-500ML; Sigma-Aldrich, St. Louis, MO, USA). Liquid–liquid interfaces were prepared under sterile conditions, immediately before the initiation of confluent culture.

### Culture conditions of MDCK cells

Cryopreserved MDCK cells (19D017; European Collection of Authenticated Cell Cultures, UK) were cultured in conventional 100-mm dishes (3020-100; AGC TECHNO GLASS, Shizuoka, JP) at 37 °C in a humidified 5% CO_2_ incubator (MCO-170AICUVD-PJ; PHC, Tokyo, JP). Upon reaching 80% confluence, cells were detached by enzymatic treatment with a 0.25 w/v% trypsin in 1 mmol/L EDTA (209–1694; FUJIFILM Wako Pure Chemical Corporation, Osaka, JP). The growth medium comprised E-MEM (051-07615; FUJIFILM Wako), 10% heat-inactivated fetal bovine serum (173012-500ML; Sigma-Aldrich), 2 mM l-glutamine (073-05391; FUJIFILM Wako), 100 units/mL penicillin, 100 μg/mL streptomycin (168-23191; FUJIFILM Wako), and 1 × MEM nonessential amino acids (139-15651; FUJIFILM Wako).

Confluent culturing was performed on fibronectin-coated or non-coated liquid–liquid or solid–liquid interfaces. Viable cells were seeded at a density of 5.3 × 10^4^ cells/cm^2^ in 24-well plates and maintained at 37 °C in a humidified 5% CO_2_ incubator. The growth medium was changed every 2 days. Culture time, *t*, was determined from the time after cell seeding in 24-well plates.

### Preparation of pinned droplets for time-lapse monitoring

To comprehend cell behaviors on liquid–liquid interfaces during maturation, we conducted time-lapse observation of MDCK cells on pinned droplets of Novec-7500, which can be captured under a microscope. As shown in Supplementary Fig. S2, fluorinated glass slides were prepared for confluent culture on pinned droplets of Novec-7500. The glass slides (10 mm × 10 mm) were treated with 10% aqueous potassium hydroxide after degreasing with 70% ethanol. After washing with ultrapure water to achieve pH 7.0, the treated glass slides were silanized on one side only with trichloro(3, 3, 4, 4, 5, 5, 6, 6, 7, 7, 8, 8, 8-tridecafluorooctyl)silane (353-23641; FUJIFILM Wako). The silanized glass slides, referred to as fluorinated glass slides, were autoclaved after washing with 99.5% ethanol. The fluorinated glass slides were aseptically placed in 24-well plates, and then 1 mL PBS was carefully introduced into each culture vessel. 100 μL Novec7500 was slowly added on each fluorinated glass slide in each well of the 24-well plate. Subsequently, 1 mL of 40 μg/mL fibronectin solution was added to each culture vessel (resulting in a final concentration of 20 μg/mL) and incubated for 1 h at room temperature. Following four washes with PBS, the surfaces were washed with E-MEM, which was later replaced by E-MEM containing 10% FBS (173012-500ML; Sigma-Aldrich). After removing E-MEM containing 10% FBS, MDCK cells were seeded at a density of 5.3 × 10^4^ cells/cm^2^ in 24-well plates, which were subsequently placed on an image analyzer (CM20; Olympus, Tokyo, JP) at 37 °C in a humidified 5% CO_2_ incubator (MCO-170AICUVD-PJ; PHC) for time-lapse imaging (Supplementary Fig. S1B).

It is crucial to consider temperature fluctuations may affect focus drift and must be accounted for. Additionally, to minimize mechanical effects on cells at liquid–liquid interfaces, it is advisable to use a microscope with a moving camera rather than a motorized stage during image capture. Thus, time-lapse images of MDCK cells were captured in 24-well plates using the image analyzer (CM20; Olympus) equipped with a 4 × objective lens, and contrast images suitable for epi-oblique illumination were captured every 15 min for 20 h (Supplementary Fig. S1B).

### Immunofluorescence staining

Fluorescence staining of ZO-2 protein (ab224314; Abcam, Cambridge, UK), F-actin, and cell nuclei of MDCK cells was performed. Briefly, MDCK cells washed with PBS were fixed with 2% paraformaldehyde (09154-85; nacalai tesque, Kyoto, JP) for 15 min at 4 °C and permeabilized by incubation in 0.05% Triton X-100 (12967-32; nacalai tesque) for 30 min at 4 °C. Nonspecific antibody binding was blocked by treating cells with Block Ace (UK-B40; Dainippon Sumitomo Pharma, Osaka, JP) overnight at 4 °C. Subsequently, cells were incubated with ZO-2 primary antibodies (1:200 dilution) overnight at 4 °C. After washing with PBS, MDCK cells were incubated with Alexa Fluor 488-conjugated goat anti-rabbit IgG (ab150077; Abcam), 4′,6-diamidino-2-phenylindole (DAPI, 342-07431; DOJINDO, Kumamoto, JP), and Alexa Fluor 594 phalloidin (A12381; Invitrogen, Waltham, MA, USA) for 3 h at room temperature. Fluorescence images were captured using a fluorescence microscope (BZ-X800; Keyence, Osaka, JP) with a 10 × objective lens (Nikon, Tokyo, JP), and the images of cell nuclei on the entire surface in the 24-well plate were captured using a 4 × objective lens. Fluorescence signal intensities were obtained via excitation at corresponding wavelengths of 358, 488, and 594 nm.

### Quantitative analysis of maturation process based on nucleus density and TJ formation

The data analysis procedure for nucleus density and the extent of TJ disruption is schematically outlined in Supplementary Fig. S3. To measure the nucleus density in regions of interest (ROI), 1.0 mm × 1.0 mm ROIs were randomly selected in a culture vessel, across three independent experiments. The staining images of nuclei and ZO-2 were analyzed using the ImageJ software^20^. Each ROI (1 mm^2^) for nucleus staining or ZO-2 was divided into 25 local squares (0.2 mm × 0.2 mm). The local cell density, *X*_LN_, was calculated from the total number of nuclei in each local square. The frequency of *X*_LN_ occurrence in each local square against the total number of local squares, *F*_x_, was estimated. To validate and quantify the relationship between nucleus density and the extent of TJ formation during maturation, MDCK cells were categorized into ZO-2-positive and ZO-2-negative groups, according to the criteria outlined in Supplementary Fig. S3B.

To ascertain the relationship between the established homogeneity index and the extent of maturation, the frequency of ZO-2-positive cells, *F*_Z_, and the ratio of nucleus density in a local square to the mean value of nucleus density, ${X}_{\text{LN}}/\overline{{X }_{\text{LN}}}$, were calculated from the data in ROIs under each culture condition. These data were used to determine the homogeneity index, *H*_LN_, representing values obtained by subtracting the standard deviation of ${X}_{\text{LN}}/\overline{{X }_{\text{LN}}}$ in each image (1 mm × 1 mm) from 1. Subsequently, *F*_Z_ and *H*_LN_ values were compared.

### Statistical analysis

Data were expressed as mean ± standard deviation, and group comparisons were analyzed using one-way analysis of variance (ANOVA) followed by the Tukey–Kramer post-hoc test. Statistical significance was established at *p < 0.05 and **p < 0.01.

## Results

### Behavioral characterization of MDCK cells on liquid–liquid interfaces

Time-lapse observation was performed for MDCK cells cultured on fibronectin-coated or non-coated liquid–liquid or solid–liquid interfaces (Supplementary Movie S1). After seeding, cells cultured on a fibronectin-coated liquid–liquid interface (Supplementary Movie S1D) and those on fibronectin-coated or non-coated solid–liquid interfaces (Supplementary Movie S1A,B), exhibited elongated shapes and active migration. As the culture time progressed, cells cultured on both fibronectin-coated liquid–liquid (Supplementary Movie S1D) and solid–liquid interfaces (Supplementary Movie S1A,B), reached a confluent state through continued active migration and division. In contrast, cells cultured on the non-coated liquid–liquid interface organized into island-like cell populations or cell-free-areas in the culture vessel, with cells in island-like regions showing reduced migration (Supplementary Movie S1C). As the culture time progressed further, the island-like regions expanded through cell growth (Supplementary Movie S1C). These results suggest that cell behaviors, such as migration and growth, serves as important cues for the maturation of epithelial cells on liquid–liquid interfaces.

**Figure 1:**
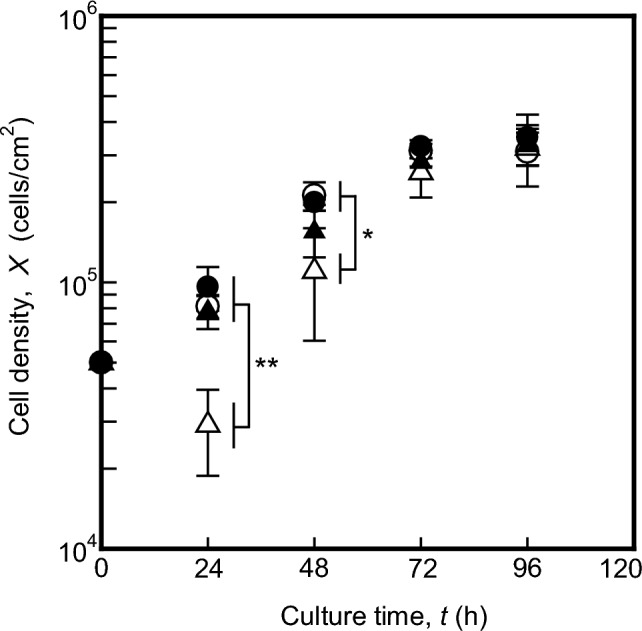
Time course of cell density of MDCK cells cultured on fibronectin-coated or non-coated liquid–liquid or solid–liquid interfaces. As shown in Supplementary Fig. S1, liquid–liquid interfaces in 24-well plate were prepared under sterile conditions, immediately before the initiation of confluent culture. Viable cells were seeded at a density of 5.3 × 10^4^ cells/cm^2^ in 24-well plates, and the cells in each culture vessel were counted at *t* = 24, 48, 72, 96 h. Open circles or triangles represent fibronectin non-coated solid–liquid or liquid–liquid interfaces; closed circles or triangles represent fibronectin-coated solid–liquid or liquid–liquid interfaces. Data were obtained from three independent cultures. Statistical significance for Student’s t-test was set at *p < 0.05; or **p < 0.01.

To understand the temporal profile of cell growth during maturation on various culture surfaces, the density of cells cultured on liquid–liquid and solid–liquid interfaces in the culture vessel were monitored until *t* = 96 h (Fig. 1). At *t* = 24 h, the seeding density of cells cultured on non-coated liquid–liquid interfaces initially decreased, but these values increased over time. By *t* = 72 h, the number of cells across all culture conditions had increased to 2.6 × 10^5^–3.2 × 10^5^ cells/cm^2^, resembling values as at *t* = 96 h and indicating contact inhibition. These results indicate that the growth of cells cultured on liquid–liquid interfaces is comparable to that of cells cultured on solid–liquid interfaces.

**Figure 2:**
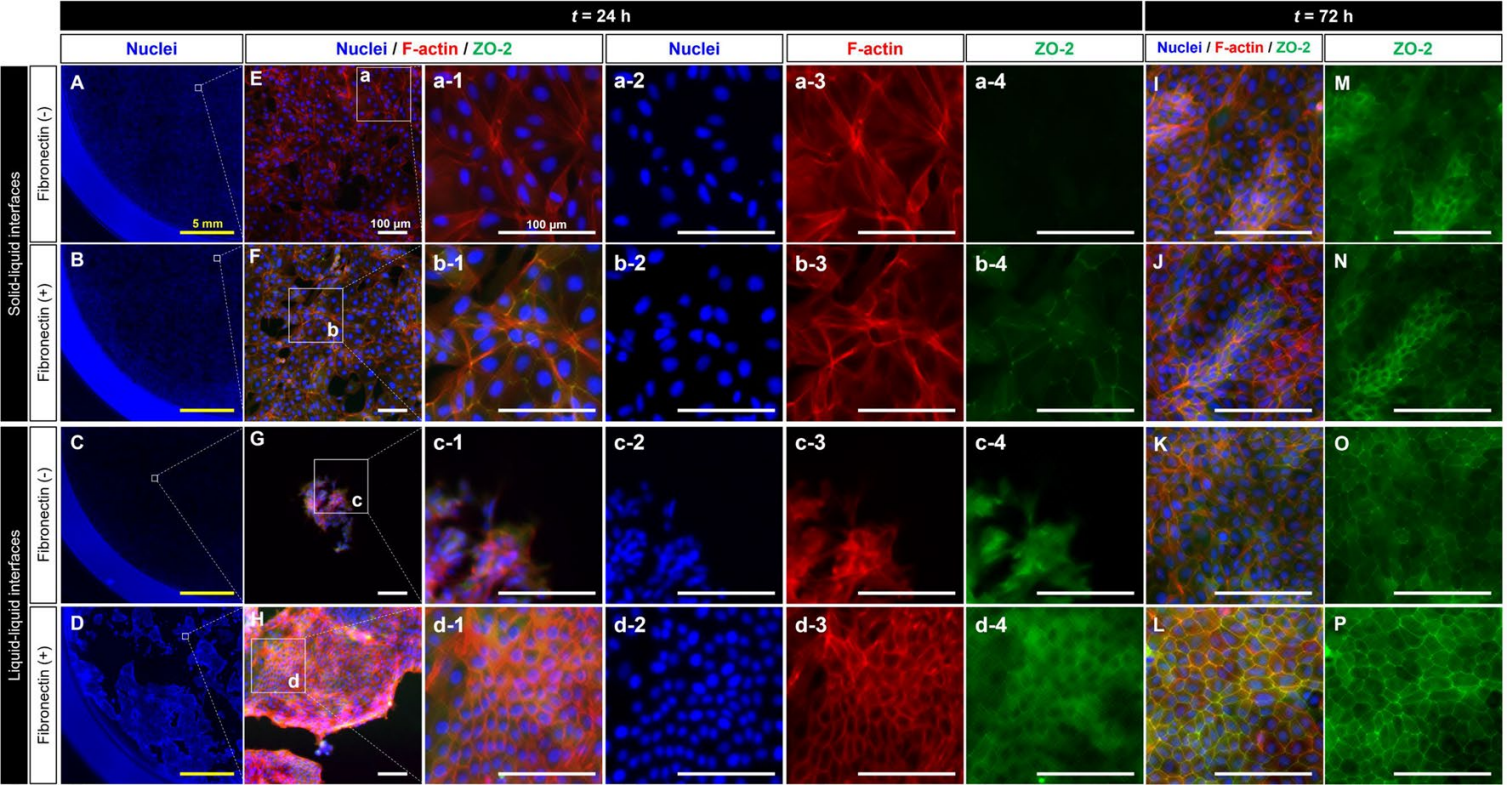
Immunostaining images of cell nuclei (DAPI, blue), F-actin (phalloidin, red) and tight junction protein (ZO-2, green) in MDCK cells cultured on fibronectin-coated or non-coated liquid–liquid or solid–liquid interfaces at *t* = 24 h and *t* = 72 h. Panels (**a-1**–**a-4**,**b-1**–**b-4**,**c-1**–**c-4**, **d-1**–**d-4**) show the enlargements of demarcated boxed areas in panels E–H. The scale bars indicate 5 mm (yellow) or 100 μm (white).

**Figure 3:**
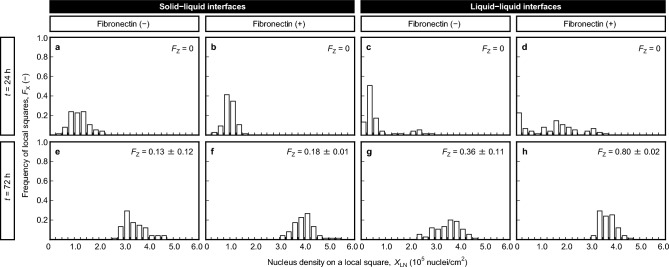
Frequencies of local squares, *F*_X_, against nucleus density on a local square, *X*_LN_, of MDCK cells cultured on fibronectin-coated or non-coated liquid–liquid or solid–liquid interfaces at *t* = 24 h (**a**–**d**) and *t* = 72 h (**e**–**h**). Data were obtained from 75 local squares in three independent cultures.

**Figure 4:**
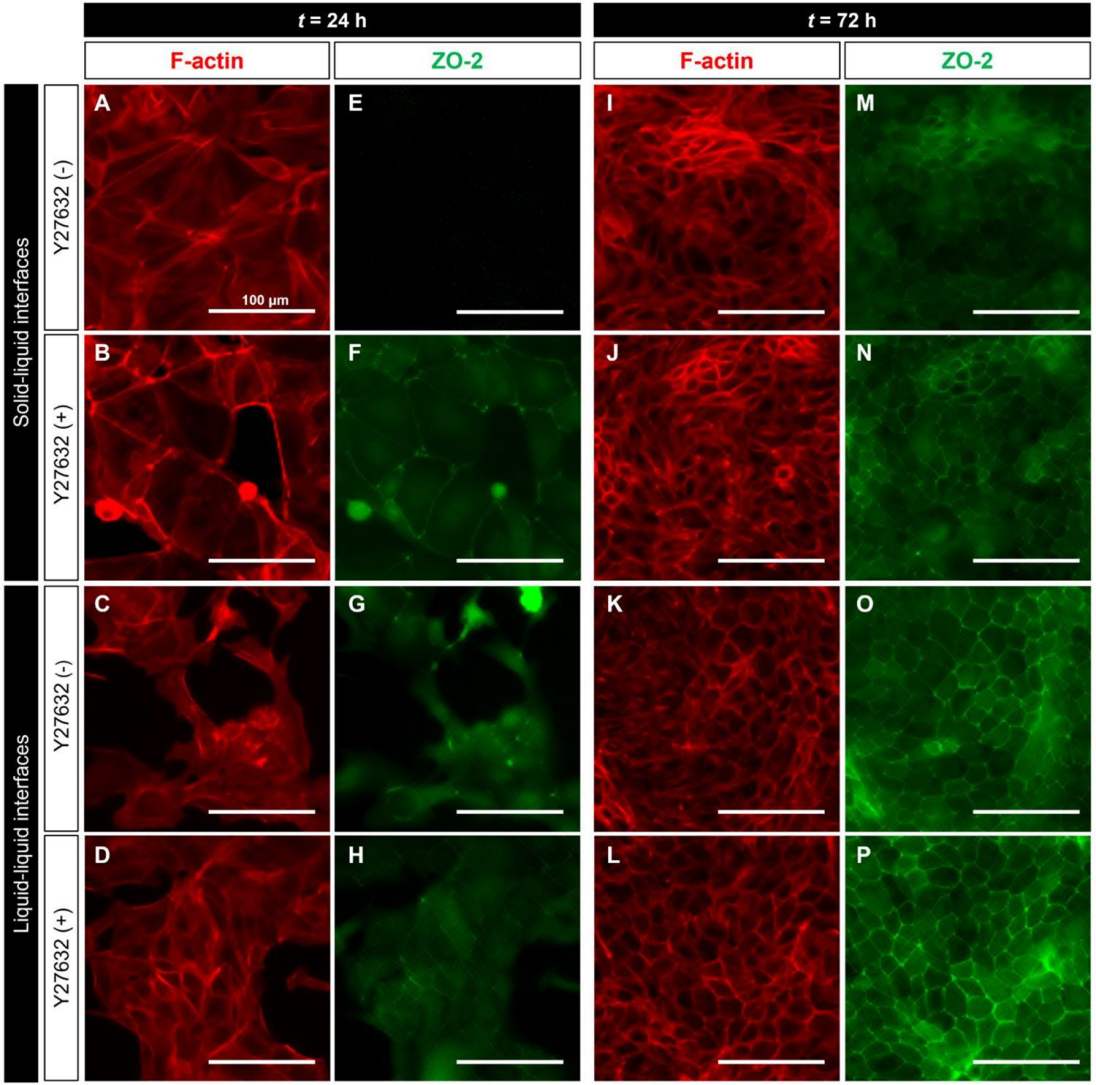
Immunostaining images of F-actin (phalloidin, red) and tight junction protein (ZO-2, green) in MDCK cells cultured with or without the Rho-dependent protein kinase inhibitor Y27632 on liquid–liquid or solid–liquid interfaces at *t* = 24 h and *t* = 72 h. MDCK cells were exposure to culture medium with or without Y27632 (10 μM) at* t* = 0–48 h. The scale bars indicate 100 μm.

To characterize MDCK cells during maturation, immunostaining for nuclei, F-actin, and ZO-2 of MDCK cells at *t* = 24 h and 72 h was performed, and immunostaining images were randomly captured in the culture vessel (Fig. 2). Additionally, the entire images of the culture vessel were captured to understand the spatial distribution of MDCK cells on the culture surfaces in each culture condition. Figures 2a–d show the immunostaining images of cell nuclei in a quarter of the culture vessel at *t* = 24 h. Notably, when cultured at the solid–liquid interface (Fig. 2A,B,E,F), the cells did not form island-like populations, and the cell densities were low in both conditions (Fig. 2a-2,b-2). Actin stress fibers were abundant in cells cultured on non-coated solid–liquid interfaces (Fig. 2a-3), and these cells did not express ZO-2 (Fig. 2a-4). When cultured on solid–liquid interfaces coated with fibronectin, stress fiber formation was inhibited (Fig. 2b-3), and ZO-2 localization was observed in a portion of the cellular membrane (Fig. 2b-4). In contrast, cells cultured on fibronectin-coated liquid–liquid interfaces formed an island-like population (Fig. 2D), exhibiting a high-density state (Fig. 2d-2). The size of the island-like population on the fibronectin-coated liquid–liquid interfaces was larger than that of the island-like population on the non-coated liquid–liquid interfaces (Fig. 2G,H). Cells cultured on liquid–liquid interfaces with high population density and reduced stress fiber formation showed ZO-2 localization in the cytoplasm (Fig. 2c-2,c-3,c-4,d-2,d-3,d-4). Ultimately, at *t* = 72 h, the polygonal cells in the high-density region had a well-defined ZO-1 line surrounding the cell (Fig. 2L,P). Furthermore, the colocalization of ZO-2 and F-actin (Fig. 2L) indicated the stabilization of cell–cell adhesion. These results suggest that the density and distribution of cells, as a consequence of cell–cell communication through actin network, are related to the maturation of epithelial cells in confluent states.

### Relationship between maturation extent and cell distribution in the culture vessel

To quantitatively investigate the relationship between the extent of TJ formation and local cell density, we determined the frequencies of local squares, *F*_X_, against nucleus density on a local square, *X*_LN_, of MDCK cells cultured on fibronectin-coated or non-coated liquid–liquid or solid–liquid interfaces at *t* = 24 h and 72 h (Fig. 3). Data were obtained from MDCK cells in triplicate experiments for each culture condition. The average values of cells cultured on non-coated and fibronectin-coated solid–liquid interfaces were $\overline{{X }_{\text{LN}}}$ = 1.0 × 10^5^ ± 0.22 × 10^5^ nuclei/cm^2^ and $\overline{{X }_{\text{LN}}}$ = 1.2 × 10^5^ ± 0.38 × 10^5^ nuclei/cm^2^, respectively (Fig. 3a,b). With elapsed culture time, the *X*_LN_ values increased in both culture conditions, ranging from *X*_LN_ = 2.5 × 10^5^ nuclei/cm^2^ to *X*_LN_ = 5.5 × 10^5^ nuclei/cm^2^ at *t* = 72 h (Fig. 3e,f). This indicates the presence of uneven cell distribution in the culture vessel. The *F*_Z_ values, reflecting broad distributions of *X*_LN_ values in both non-coated and fibronectin-coated culture conditions, were *F*_Z_ = 0.13 ± 0.12 and *F*_Z_ = 0.18 ± 0.01, respectively (Fig. 3e,f). In contrast, when cultured on fibronectin-coated liquid–liquid interfaces, a broad distribution of *X*_LN_ was observed, ranging from *X*_LN_ = 0 to *X*_LN_ = 3.8 × 10^5^ nuclei/cm^2^ (Fig. 3d). The frequency of *X*_LN_ = 0 on non-coated liquid–liquid interfaces was the highest (Fig. 3c), indicating numerous areas without cells. However, in areas where cells adhered to the liquid interface, the cell density was high, ranging from *X*_LN_ = 2.0 × 10^5^  nuclei/cm^2^ to *X*_LN_ = 3.0 × 10^5^ nuclei/cm^2^ at *t* = 24 h (Fig. 3c). At *t* = 72 h, the *X*_LN_ distribution on fibronectin-coated liquid–liquid interfaces became narrower than that on non-coated liquid–liquid interfaces, and the *F*_Z_ values reached their peak (*F*_Z_ = 0.80 ± 0.02). Collectively, these findings suggest that both high density and even distribution of cells in the culture vessel promote TJ formation, subsequently facilitating the maturation process.

### Facilitation of uniform maturation through cell behaviors on liquid–liquid interfaces

Y27632, a specific inhibitor for Rho-dependent protein kinase (ROCK), induces cell survival and suppression of stress fiber formation^21,22^. Therefore, in this study, we investigated the effect of the behavior of epithelial cells on their maturation on non-coated liquid–liquid or solid–liquid interfaces when exposed to media with and without 10 μg/mL Y27632 at *t* = 0–48 h (Fig. 4). At *t* = 24 h, exposing cells cultured on solid–liquid interfaces to Y27632 induced the suppression of stress fiber formation (Fig. 4B), leading to TJ formation (Fig. 4F). However, at *t* = 72 h, cells cultured on solid–liquid interfaces in both conditions caused F-actin disassembly (Fig. 4I,J), resulting in TJ disruption (Fig. 4M,N). In contrast, cells cultured on liquid–liquid interfaces in both culture conditions became highly dense (Fig. 4C,D), with ZO-2 localization in the cytoplasm (Fig. 4G,H). Similar to solid–liquid interface culture, when not exposed to Y27632, cells cultured on liquid–liquid interfaces at *t* = 72 h exhibited F-actin disassembly and TJ disruption (Fig. 4K,O). However, when exposed to Y27632, cells cultured on liquid–liquid interfaces at *t* = 72 h formed the actin belt (Fig. 4L), and ZO-2 colocalized with F-actin, indicating the stabilization of TJ formation at the plasma membrane (Fig. 4L,P). Interestingly, the staining image trend of F-actin and ZO-2, when exposed to Y27632, resembled that seen in cultures on fibronectin-coated surfaces (Figs. 2, 4).

**Figure 5:**
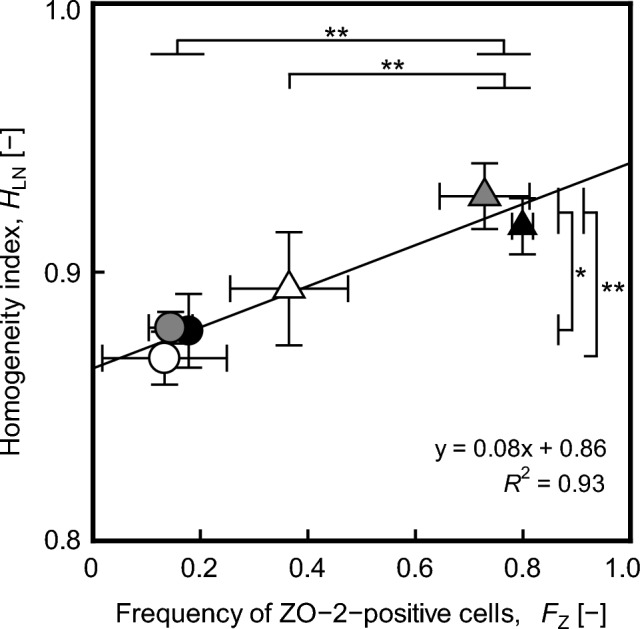
Correlation between homogeneity index, *H*_LN_, and frequency of ZO-2-positive cells, *F*_Z,_ values of MDCK cells cultured on liquid–liquid or solid–liquid interfaces at* t* = 72 h. Liquid–liquid interfaces in 24-well plate were prepared under sterile conditions, immediately before the initiation of confluent culture (Supplementary Fig. S1). Viable cells were seeded at a density of 5.3 × 10^4^ cells/cm^2^ in 24-well plates. The cells cultured on fibronectin-coated or non-coated liquid–liquid or solid–liquid interfaces. In culture condition of Y27632-exposure, the cells on non-coated liquid–liquid or solid–liquid interfaces were cultured in media with and without 10 μg/mL Y27632 at *t* = 0–48 h. From* t* = 48 h, the cells were cultured in media without 10 μg/mL Y27632. The staining images of nuclei and ZO-2 at *t* = 72 h were analyzed using the ImageJ software. The plots show *H*_LN_ and *F*_Z_ values of cultures grown on solid–liquid interfaces (shaded circles) or liquid–liquid interfaces (shaded triangles) when exposed to Y27632, as well as in cultures grown on non-coated solid–liquid interfaces (an opened circle), non-coated liquid–liquid interfaces (an opened triangle), fibronectin-coated solid–liquid interfaces (a closed circle), and fibronectin-coated liquid–liquid interface (a closed triangle) at* t* = 72 h. Exposure of cells to Y27632 was performed at *t* = 0–48 h. Data were obtained from three independent experiments. Statistical significance was determined via one-way analysis of variance (ANOVA) followed by Tukey–Kramer post-hoc test (**p < 0.01; *p < 0.05).

To further elucidate the correlation between TJ formation and even cell distribution in the culture vessel, the analysis was performed according to the procedure outlined in Supplementary Fig. S3C. Specifically, we examined the correlation between the *H*_LN_ and *F*_Z_ values of MDCK cells cultured on fibronectin-coated or non-coated liquid–liquid or solid–liquid interfaces at *t* = 72 h to achieve high cell density. The homogeneity index, *H*_LN_, serves as an indicator of the even distribution of cells on the surfaces in each culture vessel. Notably, both the *H*_LN_ and *F*_Z_ values of cells cultured on the fibronectin-coated liquid–liquid interface, as well as of Y27632-exposed cells, were significantly higher than those cultured on the non-coated liquid–liquid interfaces (Fig. 5). Across all culture conditions, the *F*_Z_ and *H*_LN_ values were closely correlated (Fig. 5) (*R*^2^ = 0.93), suggesting that achieving an even distribution of cell nuclei via cell–cell interaction is crucial for maintaining TJ formation during the late phase of maturation.

## Discussion

**Figure 6:**
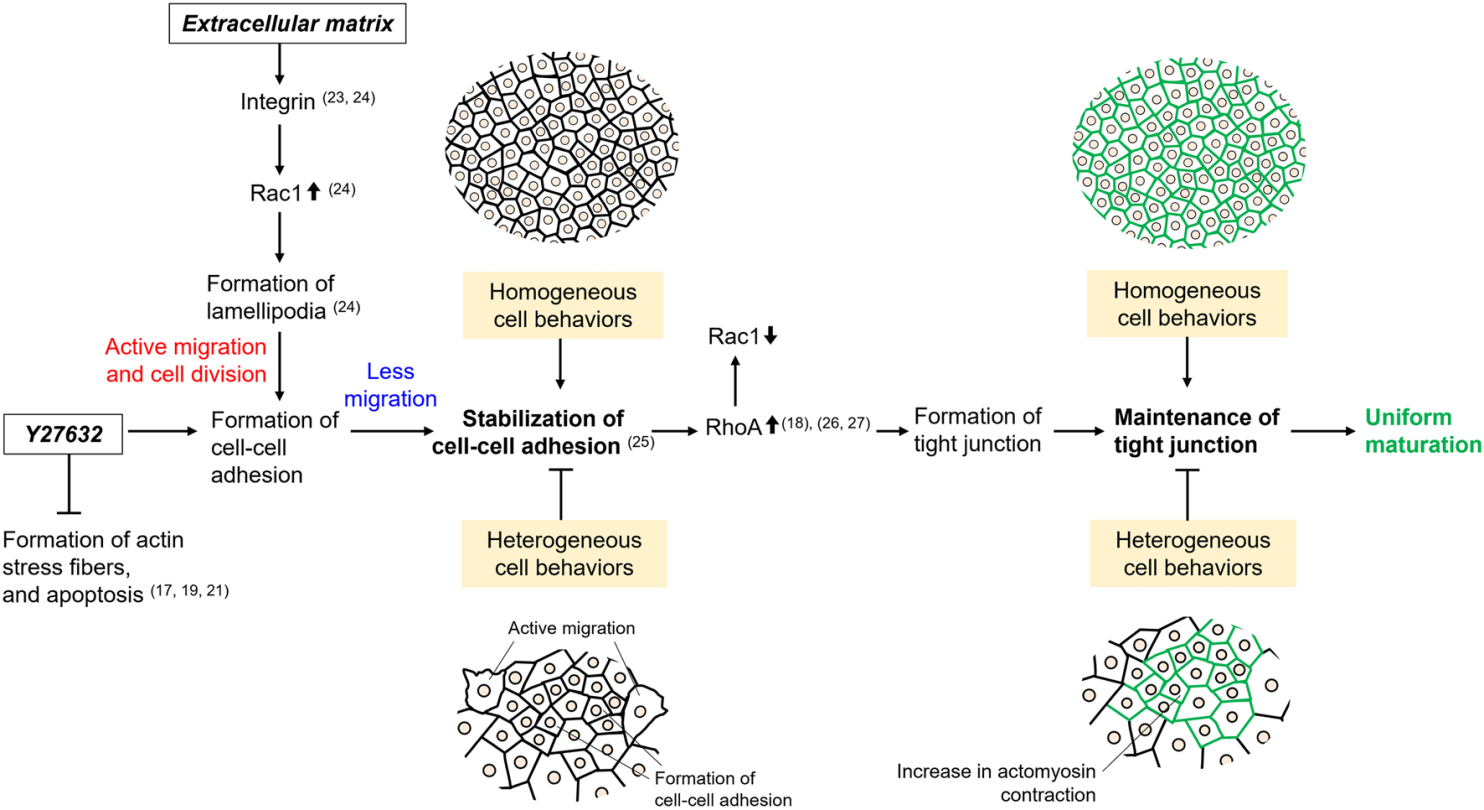
Schematic diagram of a possible mechanism of epithelial cell maturation based on the behavior of MDCK cells grown on liquid–liquid or solid–liquid interfaces.

Cell behavior plays a critical role in the maturation of epithelial cells. In this study, we examined the time-dependent profiles of cell behavior during the maturation of epithelial cells cultured on liquid–liquid interfaces. Our results indicated that the formation of tight junctions was uniformly facilitated through the behavior of MDCK cells on the liquid–liquid interface. As shown in Fig. 6, the binding of integrin β1 to fibronectin is a critical step in cell migration and growth^23,24^. Danen et al.^24^ reported that the binding of integrin β1 to fibronectin increased Rac1 activity through autophosphorylation of focal adhesion kinase (FAK), causing active migration through lamellipodia formation. As the formation of intercellular adhesions progresses, cell migration decreases, contributing to the stabilization of cell–cell adhesion. The binding of p120 catenin to E-cadherin, localized to the cell membrane, is known to inhibit endocytosis, leading to the stability of cadherin-catenin cell–cell adhesion complexes^25^. RhoA activation induces myosin activation through phosphorylation of the myosin II light chain, leading to the assembly of TJ proteins^26^. Cytosolic RhoA-GDP forms a complex with p120 catenin, thereby inhibiting RhoA activation^27^, suggesting that RhoA inhibition is a crucial event in the early phase of maturation, while RhoA activation is likely important in the late phase of maturation. ROCK, a downstream effector of RhoA, plays an essential role in the formation of both the F-actin cytoskeleton and TJs in cells during maturation^14,15,28^. In the present study, the exposure of cells to Y27632 facilitated TJ formation in epithelial cells cultured on liquid–liquid interfaces. This maturation trend was similar to that observed in culture conditions with fibronectin-coated liquid–liquid interfaces. Considering these findings, it is highly likely that the changes in cell migration and growth led to uniform maturation in epithelial cells on culture conditions of Y27632 exposure or the fibronectin-coated liquid–liquid interfaces in our study.

Two reasons why cells on the fibronectin-coated liquid–liquid interface or cells with ROCK inhibitor on liquid–liquid interface uniformly achieved maturation have been considered. First, in the early phase of the maturation, the cells on culture condition the fibronectin-coated liquid–liquid interface showed active growth, resulting in the achievement of a confluent state. The second is that in the late phase of the maturation, cells on the fibronectin-coated liquid–liquid interface were identified as the cells the distinct line of ZO-2 and actin surrounded perfectly, leading to the maintenance of tight junction formation through uniform behaviors of the cells. In addition, ROCK inhibitor induced cell behaviors, similar to cells at the fibronectin-coated liquid–liquid interface. Thus, cell behavior transitions, such as growth and migration, are pivotal for achieving uniform epithelial cell maturation. Previous studies have indicated inhibition of migration of retinal pigment epithelial cells in the late phase of maturation at the solid–liquid interface with a Rac1 inhibitor, which promoted uniform maturation^12,13^. Based on this report and the results of the current study, inhibition of migration is suggested to be important for uniform maturation of epithelial cells in the late stages of maturation. In the current study, it is possible that at the liquid–liquid interface, coating the extracellular matrix on PFC or using a ROCK inhibitor facilitates uniformity in epithelial cells by inducing uniform cell behavior. Our findings suggest that the liquid–liquid interface, which is easily recovered as epithelial tissue, may be useful as a new culture strategy for epithelial tissue production.

Quantitative analysis based on microscopic images provides information on local cell behavior and characteristics in confluent states^29–31^. In the present study, time- and location-dependent profiles of individual epithelial cells in the culture vessel were analyzed to understand changes in their behavior during maturation. The ROIs were randomly selected from the entire culture vessel, and the determination of ZO-2-positive or ZO-2-negative cells was performed at the single cell level. The results suggest that regulating the behaviors of cells cultured on liquid–liquid interfaces may facilitate the uniform maturation of epithelial cells in a culture vessel. This phenomenon may be attributed to the regulation of the F-actin network in cells cultured on the liquid–liquid interface. The inhibition of ROCK by Y27632 is known to suppress cell–cell and cell–matrix interactions, which induces a shift in mechanical forces from ‘pulling’ to ‘pushing’ neighboring cells via the inhibition of the F-actin network^22,28^. This implies that mechanical interactions within the epithelial sheet are simplified in the culture vessel, which explains cell migration during the early phase of maturation in the present study. Furthermore, Raya-Sandino et al.^14^ reported that ZO-2 plays a key role in regulating Rho, Rac, and Cdc42 proteins, essential for TJ sealing. This suggests that the localization of ZO-2 in cells has important implications for the maturation process. In the present study, ZO-2 expression was detected in the cytoplasm under culture conditions of Y27632 exposure or the fibronectin-coated liquid–liquid interface, suggesting that Rac1 activation may induce the formation of cell–cell adhesion junctions and activation of cell growth. With the elapse of culture time, the distinct line of ZO-2 and actin surrounded, were distributed on culture conditions of Y27632 exposure or the fibronectin-coated liquid–liquid interface, suggesting that these culture conditions of which resulted in the stabilization of ZO-2 on the plasma membrane. Subsequently, the cells exhibited an even distribution in the culture vessel (*H*_LN_ = 0.93 ± 0.01 and *H*_LN_ = 0.92 ± 0.01), resulting in the facilitation of TJ formation in the present study (*F*_Z_ = 0.73 ± 0.08 and *F*_Z_ = 0.73 ± 0.02). Although several factors may contribute to facilitating uniform maturation during culture on liquid–liquid interfaces, the spatiotemporal control of cell migration and growth, as a consequence of cell communication, is one of the crucial events promoting uniform maturation of epithelial cells. However, it is unknown how the mechanism of their behavior is driven by mechanical forces in cell–cell interactions on liquid–liquid interfaces in the entire culture vessel. In addition, it remains unclear whether the mechanical stimulation is due to forces resulting in flow from the liquid scaffold or the viscoelasticity of the material, such as the extracellular matrix. Therefore, spatiotemporal elucidation of cell–substrate and cell–cell interactions on liquid–liquid interfaces in the entire culture vessel is required. To address the issue, further technological development is needed to develop microscopes and data processing methods that enable observation and huge image processing of cells or flow on liquid–liquid interfaces. Here, we have focused on their behavior through cell–cell interactions as a result of mechanical interactions and have proposed a breakthrough approach to ensure uniform maturation of epithelial cells at liquid–liquid interfaces. It can be concluded that culture conditions involving Y27632 exposure or a fibronectin-coated liquid–liquid interface facilitate uniform maturation of cells through the regulation of their migration and growth.

## Conclusions

MDCK cells were cultured to understand their behaviors influencing maturation on liquid–liquid interfaces in a culture vessel, employing time-lapse observation and immunostaining. Cells adhering to the fibronectin-coated liquid–liquid interface, as well as polystyrene typically used for normal cell cultures, exhibited elongated shapes, attaining a confluent state through active migration and division. The ZO-2-positive cells exhibited an even distribution of nuclei, facilitated by cell–cell interaction via F-actin network. The frequency of ZO-2-positive cells on the fibronectin-coated liquid–liquid interface was 2.2-fold higher than that on the non-fibronectin-coated liquid–liquid interface at *t* = 72 h. Similarly, the exposure of cells to Y27632 resulted in an even distribution of nuclei in the culture vessel, facilitating TJ formation. Our findings suggest that in vitro confluent culture using liquid materials can offer a novel perspective on the maturation process of epithelial cells in the culture vessel, providing an approach to regulate their fate through cell behavior.

### Supplementary Information

Supplementary Information 1.

Supplementary Information 2.

Supplementary Information 3.

Supplementary Video 1.

Supplementary Information 4.

## Data Availability

The data that support the findings of this study are available from the corresponding author upon reasonable request.
