# Supplementary figures and images for: A novel strategy to facilitate uniform epithelial cell maturation using liquid–liquid interfaces
Source: Sci Rep. 2024 May 29;14:12314. doi: 10.1038/s41598-024-63115-7 (PMC11137049; doi:10.1038/s41598-024-63115-7)

## Slide 1
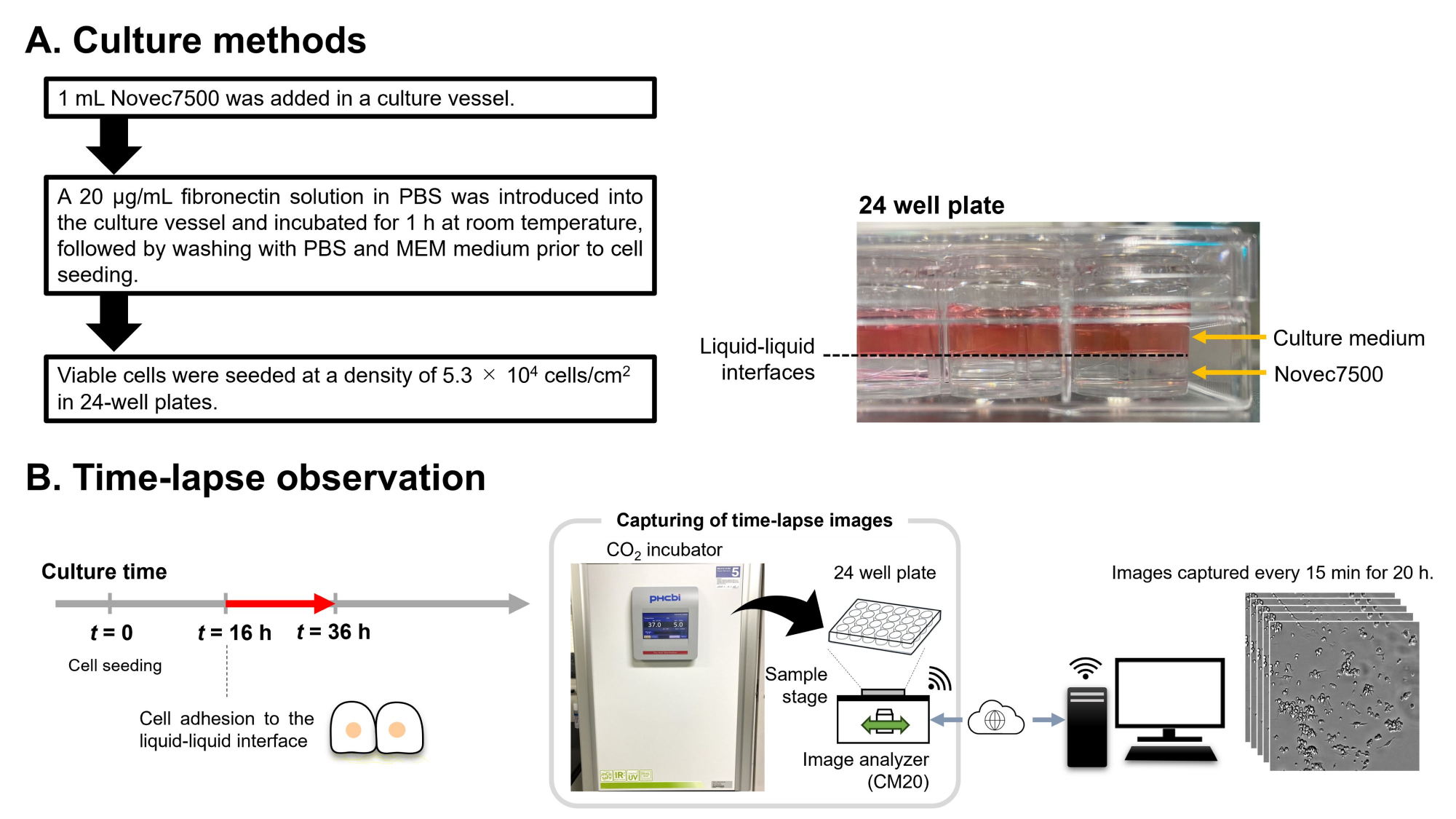

Supplement: Supplementary file 1 — Supplementary Information 1. [file 41598_2024_63115_MOESM1_ESM.pptx]

## Slide 1
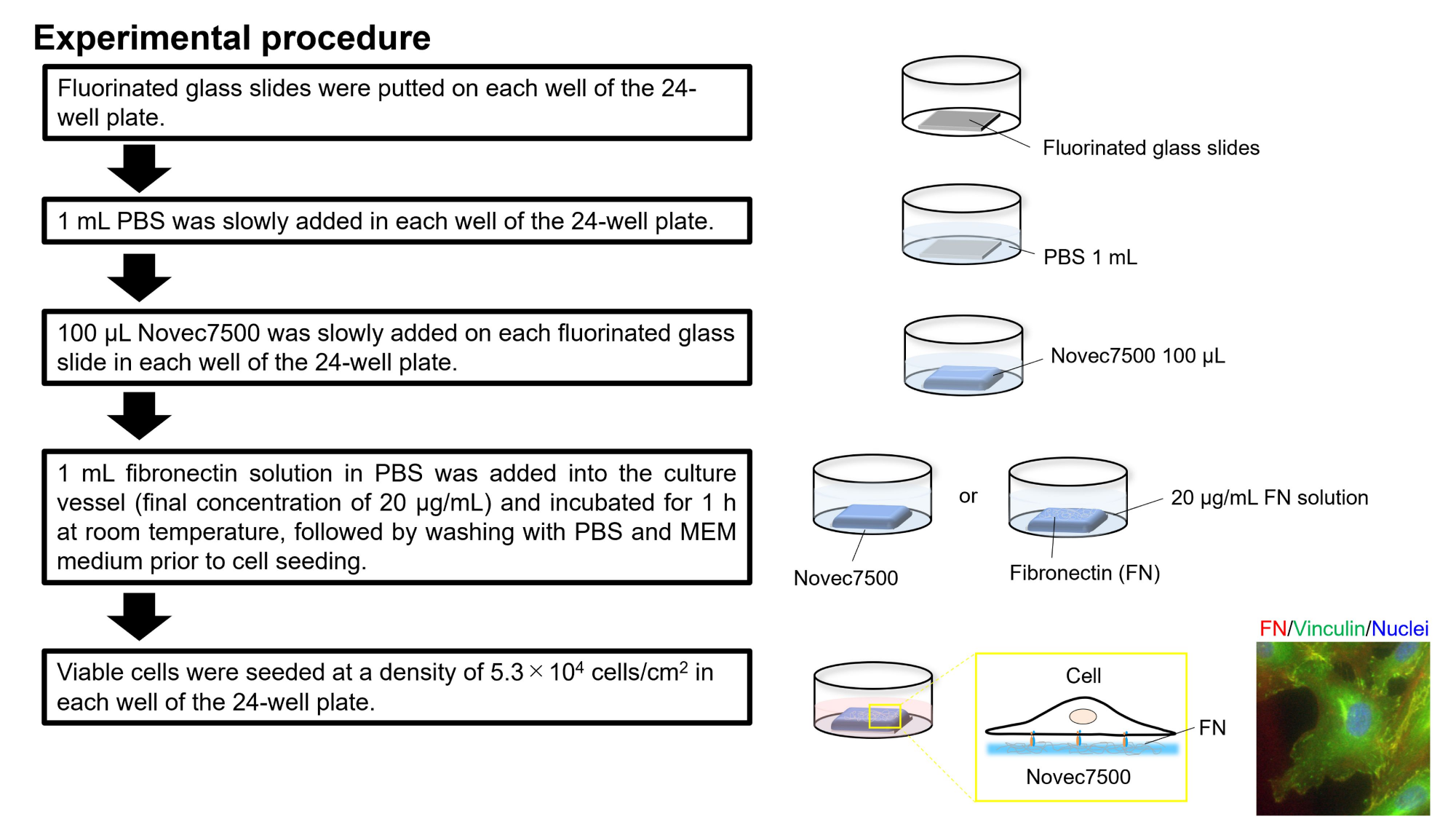

Supplement: Supplementary file 2 — Supplementary Information 2. [file 41598_2024_63115_MOESM2_ESM.pptx]

## Slide 1
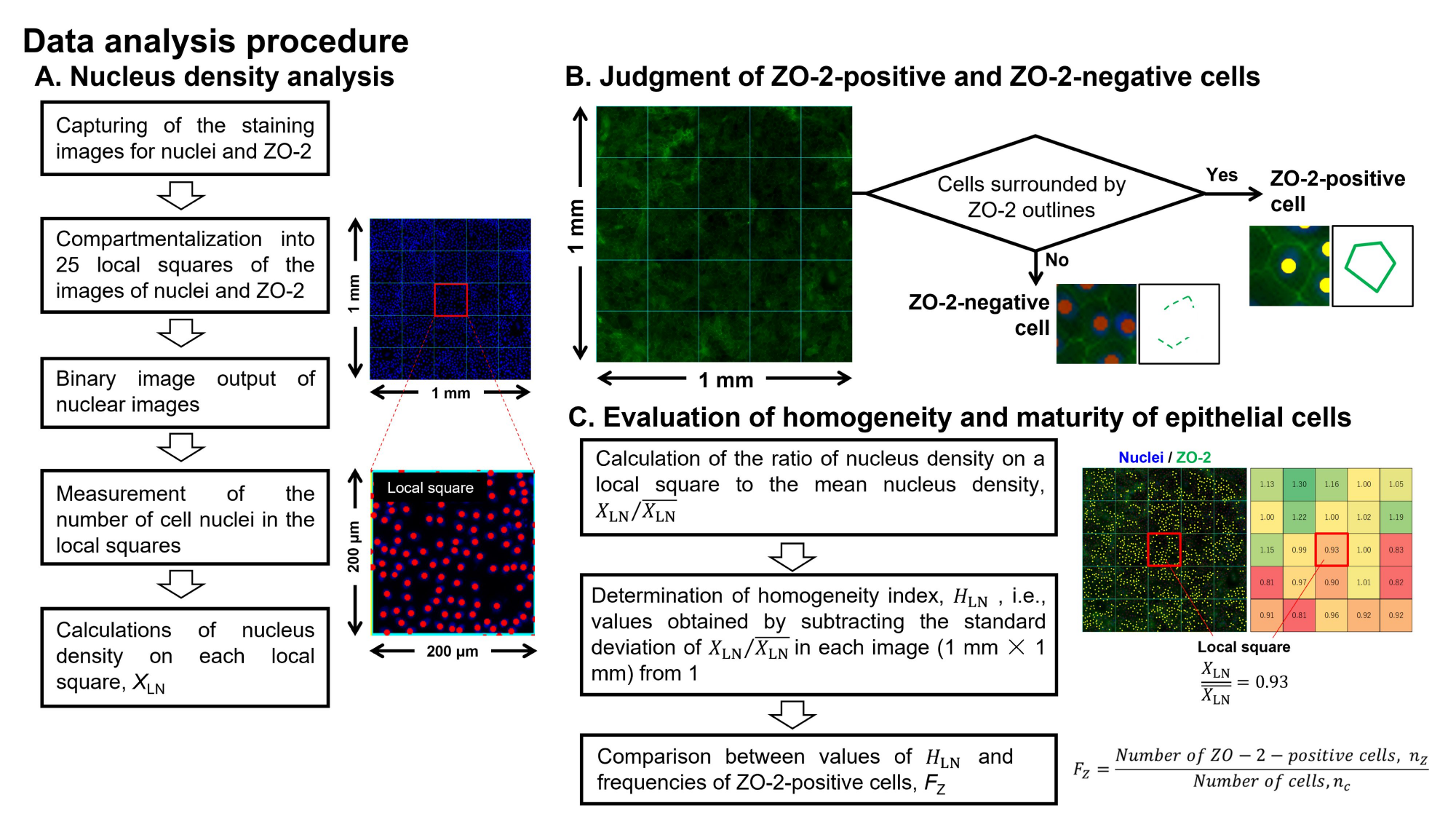

Supplement: Supplementary file 3 — Supplementary Information 3. [file 41598_2024_63115_MOESM3_ESM.pptx]
