# Supplementary material for: A novel strategy to facilitate uniform epithelial cell maturation using liquid–liquid interfaces
Source: Sci Rep. 2024 May 29;14:12314. doi: 10.1038/s41598-024-63115-7 (PMC11137049; doi:10.1038/s41598-024-63115-7)
Supplement: Supplementary file 5 — Supplementary Information 4. [file 41598_2024_63115_MOESM5_ESM.docx]

**Online supplementary material**

Figure S1: Experimental procedure to understand their behaviours of MDCK cells on liquid–liquid interface.

Figure S2: Experimental procedure of MDCK cells on pinned droplets for time-lapse monitoring.

Figure S3: Data analysis procedure based on the staining images of cell nuclei and ZO-2 in MDCK cells during maturation.

Movie S1: Time-lapse observations of MDCK cells cultured on fibronectin coated, or non-coated, the liquid–liquid or the solid-liquid interfaces at t = 16–36 h.
